# Integration of mental health into primary care in low- and middle-income countries: the PRIME mental healthcare plans

**DOI:** 10.1192/bjp.bp.114.153668

**Published:** 2016-01

**Authors:** Crick Lund, Mark Tomlinson, Vikram Patel

**Affiliations:** **Crick Lund**, BA, BSocSci (Hons), MA, MSoSci (ClinPsych), PhD, Alan J Flisher Centre for Public Mental Health, Department of Psychiatry and Mental Health, University of Cape Town, Cape Town, South Africa, and Centre for Global Mental Health, Institute of Psychiatry, Psychology and Neuroscience, King's College London, UK; **Mark Tomlinson**, BA, BA (Hons), MA (ClinPsych), PhD, Alan J Flisher Centre for Public Mental Health, Department of Psychology, Stellenbosch University and Department of Psychiatry and Mental Health, University of Cape Town, Stellenbosch, South Africa; **Vikram Patel**, PhD, MRCPsych, FMedSci, Centre for Global Mental Health, London School of Hygiene and Tropical Medicine, London, Centre for Chronic Conditions and Injuries, the Public Health Foundation of India, New Delhi, and Sangath Centre, Goa, India

## Abstract

This supplement outlines the development and piloting of district mental healthcare plans from five low- and middle-income countries, together with the methods for their design, evaluation and costing. In this editorial we consider the challenges that these programmes face, highlight their innovations and draw conclusions.

In this supplement to the *British Journal of Psychiatry*, we present district mental healthcare plans from five low- and middle-income countries (LMIC) participating in the PRogramme for Improving Mental health carE (PRIME). These articles are accompanied by the design, evaluation and costing methodologies for the PRIME mental healthcare plans (MHCPs). This supplement is the culmination of more than 3 years of work, and collaboration between a range of academic institutions, non-governmental organisations (NGOs), ministries of health and the World Health Organization (WHO).^1^

The challenge of scaling up mental health services in LMIC is less one of what to implement, than one of how to implement. There is already robust evidence for a range of cost-effective interventions,^2,3^ but little evidence on how these may be delivered in diverse low-resource settings. Through partnerships with a range of stakeholders in the PRIME countries, we embarked on a rigorous process of initially developing a framework for district MHCPs, conducting formative research to gather data needed to populate this framework, pilot testing the implementation of the plans and developing methodologies to evaluate their costs (inputs), process and outcomes. Our hope is that this supplement will be a resource for ministries of health, NGOs and researchers wishing to implement and scale up mental health services in other LMIC. Although the data that we present in this supplement emanate from diverse cultural, social and economic settings, the overall framework for district MHCPs is shared by all settings, as are the methodologies for design, costing and evaluation. We therefore share these findings in order to stimulate engagement from a range of local, national and international agencies, wishing to commit themselves to narrowing the enormous treatment gap for mental healthcare in LMIC.

The purpose of these articles and the work of PRIME is to answer questions regarding the implementation and scaling up of packages of care for mental disorders in LMIC.^1^ Our initial situation analysis of the five district demonstration sites in the PRIME countries documented the challenging environments in which we have chosen to work.^4^ Some of the challenges facing the implementation of packages of care in these settings include the lack of priority for mental health over other health needs of the countries; high levels of poverty and social deprivation; diverse cultural settings; broader health system challenges such as weak coordination between national and local authorities, inadequate medication supply and weak health management information systems; burden on the front-line healthcare workers; shortages of human resources; inflexible bureaucracy; lack of accountability among the implementing agencies; and demand-side constraints, including limited community awareness, high levels of stigma and discrimination against people living with mental illness, and diverse explanatory models, which may influence the acceptability and uptake of services.

## Overview of the articles

We present the findings of this new research in nine articles. From Ethiopia, Fekadu *et al*^5^ describe the MHCP for Sodo district in the Guraje zone. The plan was developed through extensive consultations with local and national stakeholders, a situational analysis, qualitative formative research, asset mapping and theory of change (ToC) workshops. The resulting mental health plan is presented at three levels: the service organisation, facility and community levels. Key to the implementation of this plan is both the provision of awareness raising and training for staff at all levels, and the establishment of ongoing support and supervision structures to enable sustainability of the plan over time. An important feature of the work in Ethiopia is continued interaction with the wider national Ministry of Health strategic plan for mental health, which envisages major scale up of mental health services in the coming years.

From India, Shidhaye *et al*^6^ present the MHCP for Sehore district in Madhya Pradesh, following a five-step development process: formative research, modelling of the packages, implementation planning, initial piloting in a single facility and expert consultation for the refinement of the plan. The Indian MHCP can be broadly divided into enabling packages and service delivery packages. The enabling packages such as programme management, capacity building and community mobilisation establish the foundation for facilitating the service delivery packages, which in turn focus on awareness for mental disorders, identification, treatment and recovery. The Indian piloting experience revealed that unless there are dedicated mental health staff inserted into the system in addition to training primary care providers in the detection and management of mental disorders, there is little change in detection and treatment practices. This finding is confirmed in other studies – most notably in Brazil^7^ and Kenya.^8^ Shidhaye and colleagues therefore show the importance of introducing a ‘vertical’ component in the form of a mental health case manager at community health centre level, in order to support the horizontal integration of mental health services in primary care, using a collaborative model of care.

From Nepal, Jordans *et al*^9^ present the MHCP for the southern district of Chitwan. The plan consists of 12 packages of care, delivered across service organisation, facility and community platforms. Similarly to other countries, the Nepal team engaged in detailed formative research,^10^ including extensive consultation with local and national stakeholders, to develop a collaborative plan with a high level of local buy-in. In addition to core features that enhance detection, culturally appropriate treatment and recovery, there are a number of unique features to the Nepal plan. These include the development of a community informant detection tool, for use by lay community informants to detect alcohol use disorders, depression, epilepsy and psychosis, using locally validated case vignettes;^11^ the provision of focused psychological treatments by community counsellors, who operate in conjunction with primary healthcare workers who do not have the time to offer these relatively time-intensive therapeutic services; and the development and piloting of a tool to assess competency of general primary healthcare workers in delivering psychological treatments and basic mental healthcare: the ENhancing Assessment of Common Therapeutic factors (ENACT) rating scale.^12^

From South Africa, Petersen *et al*^13^ present the MHCP for the Dr Kenneth Kaunda District in the North West Province. In line with the current national Department of Health primary care revitalisation policy, with plans to establish a national health insurance system, the PRIME South Africa plan focuses on the inclusion of mental health in the integrated chronic disease management platform. The PRIME team engaged in an extensive formative research and consultation process to develop the plan, and built on existing tools developed by the Department of Health and PRIME partners, such as the Primary Care 101 (PC101) clinical and training guidelines for primary healthcare workers.^14^ As in the Indian experience, piloting of the plan in one facility revealed limited detection and uptake of treatment packages for depression, alcohol use disorders and schizophrenia. Building on this experience, the paper identifies a number of strategies for addressing these challenges, which are used to strengthen the plan. These include change management workshops for facility and district managers; strengthening the mental health component of the PC101 clinical and training guideline; clarifying the roles of primary care nurses and community counsellors; and strengthening the role of community health workers in tracking and supporting service users, among others.

From Uganda, Kigozi *et al*^15^ describe the development of the MHCP in the Kamuli district. They describe the process of developing the plan, which included a situation analysis, qualitative formative research, ToC workshops and the costing tool. As with other countries, the MHCP is structured according to three levels: healthcare organisation, health facility and community level. In Uganda's case, epilepsy is added to the PRIME priority disorders of depression, alcohol use disorders and psychosis. Packages of care focus on the adapted WHO Mental Health Gap Action Programme (WHO mhGAP) intervention guide,^16^ which is being used to train primary care workers in the detection, treatment and referral of these disorders. In a similar manner to other countries, the piloting experience revealed reluctance to taking on new mental health tasks on the part of primary healthcare workers, and a request for more extensive mental health training and more frequent supervision for mental healthcare. Plans to address these shortcomings include greater community sensitisation about mental health, and more extensive training and supervision.

In the cross-country mental healthcare plan article, Hanlon *et al*^17^ synthesise the country plans, documenting the common elements and the country-specific adaptations of the plans in these diverse settings. This is an important element of the work, as it allows policy makers, researchers and implementers to distil key lessons that may be applicable in other diverse LMIC settings. Many of the differences between countries are driven by the resource context, with similarities emerging most prominently between the low-income countries (Ethiopia, Nepal and Uganda) and between the middle-income countries (India and South Africa). Despite these differences, there are many elements that are shared between countries. These include shared objectives and a common overall planning framework; the high level of participation and engagement with local stakeholders; the focus on community, health facility and health organisation levels; challenges of overburdened primary healthcare systems; and the limited impact of training without systemic changes in the form of new mental health resources, referral pathways, improved medication supply and reorientation of health facility managers.

In the ToC paper, Breuer *et al*^18^ describe the process of developing the PRIME cross-country ToC, and its adaptation and further development in each country setting. The authors document the use of ToC not only as a planning tool, but also as an evaluation tool, by describing the way in which the causal pathway set out in the ToC map enables the identification of key indicators for the successful implementation of a MHCP. This then provides an overall framework for the evaluation design of PRIME. The ToC approach offers a novel method for both the design and evaluation of MHCPs in LMIC, and the template developed by PRIME may be used by other countries or districts wishing to implement and scale up mental healthcare for a range of disorders.

The methodology paper by De Silva *et al*^19^ outlines the evaluation design for the implementation of the MHCPs across the five country sites. These include repeat community-based cross-sectional surveys to measure change in population-level contact coverage; repeat facility-based surveys to assess change in detection of disorders; disorder-specific cohorts to assess the effect on patient outcomes; and multilevel case studies to evaluate the process of implementation. The combination of these methods will allow us to answer questions that are not possible with single methods. For example, combining findings regarding contact coverage from the community surveys with clinical outcomes from the cohort studies will allow us to estimate the effective coverage of the MHCPs.

Finally in the costing paper, Chisholm *et al*^20^ set out the method for calculating the costs of implementing the MHCP in each country, over a 5- to 15-year scale-up period. Key parameters regarding the target population, prevalence of the priority disorders, resource quantities (including human resource needs and essential psychotropic medications), prices or unit costs and coverage are used to estimate costs of the MHCP for each country site. The estimated cost of scaling up an evidence-based package of care in non-specialist healthcare settings of an evidence-based package of care ranges from US$0.20 to 0.60 per capita in four out of the five districts. However, in South Africa, an upper-middle-income country where the prevailing price and quantity of healthcare service inputs are much higher, the cost per capita of delivering the specified care packages at target coverage levels approaches US$2 per capita. These findings can inform policy makers about the financial and human resource implications of implementing the MHCPs in these diverse settings. They also provide a methodology for estimating the cost of implementing integrated mental healthcare packages in other LMIC.

## Significance of the research

To the best of our knowledge, this is the first time that a variety of LMIC have devised detailed district-level plans for the integration of mental health into primary care, using a common implementation and design framework. This is a significant step forward in answering challenging questions regarding how evidence-based mental health interventions can be delivered in an integrated and culturally sensitive manner, with a high level of local stakeholder buy-in, in low-resource settings. The framework may be used and adapted by a range of other countries, in their efforts to address the large treatment gap.

The material generated by this programme includes a range of supporting materials, set out in the online supplements, that include country-specific ToC maps, elaborated country-specific mental healthcare packages, detailed cross-country comparisons of the human resource mix, various implementation tools and more details of the evaluation design for PRIME. We hope that these materials may be used and adapted by other researchers, policy makers and planners in LMIC, wishing to implement and scale up evidence-based and culturally appropriate mental healthcare.

This research has a number of limitations. Chiefly, although the articles in this supplement describe the development and evaluation methods for MHCPs, they do not set out to assess the impact of their implementation on identification and treatment in these settings. The next stage of PRIME research will include evaluation of the implementation of the MHCPs. This will include assessing changes in detection rates for depression and alcohol use disorders, changes in treatment coverage for these disorders in the district populations, and the clinical, social and economic outcomes for individuals who receive care for depression, alcohol use disorders, psychosis and epilepsy. Further research is needed on the scaling up of such treatment packages for larger populations, and the implementation of treatment packages for other priority disorders, for example disorders of childhood and adolescence.

The development and evaluation of the PRIME MHCPs is a response to calls to scale up mental health services in LMIC, expressed by researchers, practitioners, patients and policy makers from around the world. Our hope is that the plans presented in this supplement pay attention to the urgent global need to address mental health as a public health and development issue, while at the same time remaining sensitive to local needs and priorities. We welcome rigorous and critical engagement with our approach, and the strengthening of partnerships to improve the lives of people living with mental health problems, who remain among the most vulnerable and marginalised populations.
